# Evaluation of surgical outcomes and prognostic factors of second primary lung cancer based on a systematic review and meta-analysis

**DOI:** 10.1186/s12893-023-02003-9

**Published:** 2023-04-21

**Authors:** Jie Zhao, Zhenghai Shen, Yunchao Huang, Guangqiang Zhao, Wei Wang, Yantao Yang, Chen Zhou, Lianhua Ye

**Affiliations:** 1 Department of Thoracic Surgery I, The Third Affiliated Hospital of Kunming Medical University (Yunnan Cancer Hospital), Kunming, China; 2 Department of Thoracic Surgery, Taihe Hospital (Hubei University of Medicine), Shiyan, China

**Keywords:** Second primary lung cancer, Prognosis, Surgery, Meta-analysis

## Abstract

**Background:**

Although surgery has been widely applied for SPLC therapy, there is still no uniform treatment approach. Whether SPLC and primary lung cancer have similar prognostic characteristics remains controversial. Herein, based on a systematic review and meta-analysis, we aimed to enucleate the influences of diverse surgical strategies and underlying prognostic factors on the prognosis of patients with both the first primary lung cancer and SPLC underwent surgical resection.

**Methods:**

A comprehensive and systematic literature search was implemented in three databases (MEDLINE, EMBASE, and Cochrane), and eligible studies were screened following inclusion and exclusion criteria. Meanwhile, we extracted the hazard ratios (HR) together with 95% confidence intervals (CI) for each prognostic factor, either directly or indirectly, from the enrolled literature.

**Results:**

Eleven studies (published between 2000 and 2022) were included in this study, including 1,131 SPLC patients. The overall survival (OS) exhibited no difference between patients with lobectomy and sublobar resection after SPLC (HR: 0.87, 95%CI: 0.62–1.21, *P* = 0.41). The patients after completion pneumonectomy had a poor prognosis (HR: 1.85, 95% CI: 1.34–2.55, *P* < 0.01). Poor prognostic factors after SPLC surgery included synchronous SPLC (HR: 3.38, 95%CI: 1.53–7.46, *P* < 0.01), tumor diameter > 2 cm (HR: 2.44, 95%CI: 1.73–3.44, *P* < 0.01), solid predominant in CT morphology (HR: 3.08, 95% CI: 1.14–8.33, *P* = 0.03), lymph node metastasis (HR: 2.79, 95%CI: 1.40–5.56), and smoking (HR: 2.37, 95%CI: 1.08–26.82, *P* < 0.01). Tumor disease-free interval (DFI), tumor histological type, and gender had no impact on the prognosis of patients received SPLC surgery.

**Conclusions:**

Patients with SPLC, especially those with poor cardiopulmonary function reserve, should be prioritized for sublobar resection for treatment. These patients should also try to avoid completion pneumonectomy. Patients with synchronous SPLC, tumor diameter > 2 cm, solid predominant in CT morphology, lymph node metastasis, and smoking had a poor prognosis. Meanwhile, SPLC has similar prognostic characteristics with single primary lung cancer. However, the study has some limitations and more evidence is warranted to verify the findings.

## Introduction

Primary bronchial lung cancer is a malignant neoplasm with a high prevalence rate and death rate in the world and poses a grave threat to human health [1]. Patients with early non-small cell lung cancer have more than 90% of the 5-year survival rate after complete resection [2]. With the advances in imaging techniques such as positron emission tomography (PET) and high-resolution computed tomography (HRCT), concerns about lung cancer screening, as well as the improved postoperative survival rate post primary lung cancer resection, the risk of second primary lung cancer (SPLC) is elevated with the increase of follow-up time [3]. There is approximately 1%-2% incidence of SPLC after primary lung cancer resection annually [4, 5].

Through intensive investigation of SPLC, Martini and Melamed [6] first proposed the diagnostic standard of SPLC, which was modified and improved by Antakli et al. [7]. This version is generally accepted and summarized in Table 1. SPLC can be divided into two types under the diagnostic standard: synchronous SPLC (sSPLC) and metachronous SPLC (mSPLC). The eighth edition of the American Joint Committee on Cancer (AJCC) for lung cancer staging defines other nodules in different locations,and they are staged differently.Tumor nodules, positioned in the same lobe, different lobes on the same side, and the opposite lobe on the different lobes, were defined as T3M0, T4M0, as well as M1a, respectively. The staging system, under the guidelines, has been ineffective in distinguishing between SPLC and intrapulmonary metastasis (IM). These nodules are considered IM of primary lung cancer, and surgical treatment is not generally recommended. SPLC and IM have different biological behaviors and prognoses [8–10]. Currently, it is generally believed that surgery is the first-line therapy for SPLC patients with sufficient lung function reserve and without distant metastasis [11–14]. A number of previous studies have mixed SPLC and IM, resulting in vastly different results. For SPLC, none of these diagnostic criteria and guidelines suggests a specific diagnostic strategy. At present, the resection range of SPLC is still controversial [14–17]. Moreover, the overall prognosis of patients after SPLC surgery varied greatly, and the prognostic effects of diverse clinical features were also inconsistent. Nevertheless, whether SPLC and single primary lung cancer have similar prognostic characteristics remains controversial.

**Table 1:** Criteria for the definition of second primary lung cancer (SPLC)

Martini and Melamed criteria	Antakli et al. Modifications
Synchronous SPLC (sSPLC)	A. Different histological conditions
A. Tumors physically distinct and separate	B. Same histological condition with two or more of the following
B. Histological type	1. Anatomical distinct
1. Different	2. Associated premalignant lesion
2. Same, but in different segment, lobe or lung if	3. No systemic metastases
a. Origin from carcinoma in situ	4. No mediastinal spread
b. No carcinoma in common lymphatics	5. Different DNA ploidy
c. No extrapulmonary metastases at the time of diagnosis	
Metachronous SPLC (mSPLC)	
A. Histologically different	
B. Histologically identical, if	
1. Free interval between cancers ≥ 2 years, or	
2. Origin from carcinoma in situ	
3. Second cancer in different lobe or lung, but:	
a. No carcinoma in common lymphatics	
b. No extrapulmonary metastases at the time of diagnosis	

In this research, based on a systematic review and meta-analysis, we aimed to elucidate the impacts of diverse surgical approaches and various underlying prognostic factors on the prognosis of SPLC patients with both the first primary lung cancer and SPLC underwent surgical resection.

## Materials and methods

### Search strategy

This meta-analysis was implemented following the guidelines of the preferred Reporting Items for Systematic Reviews and Meta-Analyses (PRISMA) [18]. A comprehensive online search of MEDLINE, EMBASE, and Cochrane databases identified studies on the prognosis of SPLC surgery between January 2000 and August 2022. The search term combination: (second primary lung cancer OR multiple primary lung cancer OR MPLC OR SPLC OR separate primary lung cancer OR multifocal lung cancer) AND (wedge resection OR segmentectomy OR lobectomy OR sublobar resection OR pneumonectomy OR surgery OR operative).

JZ and WW independently performed study screening. The titles and abstracts of all identified publications were screened through an online search, followed by reading the full text of all preliminary screening studies. During this process, any differences encountered were discussed and resolved with the senior author (LY). This systematic review with a meta-analysis of the data from an individual patient was registered on INPLASY (INPLASY2022110047).

### Study eligibility

Publications selected for inclusion meet the following standards: (1) SPLC must be clearly defined in the article; (2) There are cases with mSPLC and/or sSPLC in the article; (3) Both the primary lung cancer and SPLC need to be surgically resected in the article; (4) Five-year overall survival (OS) rate, calculated from the start of the SPLC surgery, should be provided in the study. Publications were excluded for these reasons: (1) Letters, reviews, editorials, case reports, and conference abstracts; (2) Articles published in non-English; (3) The study included primary malignancies of other organs or IM; (4) Incomplete prognostic data, or failing to extract the the risk ratio (HR) and 95% confidence intervals (CI).

### Data acquisition and quality evaluation

Two authors (ZS and YY) carefully read the full text of the study and extracted the data independently. The extracted data consisted of the first author, study area, publication year, year of study start and end, number of patients, 5-year OS, as well as prognostic factors (gender, smoking status, histological type, tumor size, lymph node metastasis (LNM), type of surgery, and tumor CT morphology) with their HR and 95% CI.

Two authors (CZ and WW) independently completed the quality evaluation, and any disagreement was discussed and resolved with the author (LY). Following the standards recommended by the Newcastle–Ottawa Scale (NOS), the quality of selected publications was modified There were 3 domains for NOS, including patient selection (0–4 points), comparability of subjects (0–2 points), as well as clinical outcome (0–3 points). There were nine points in total for NOS scores, and studies with equal to or more than 5 points were regarded with high quality.

### Statistics

HR and standard error (SE) are utilized for data consolidation. HR and 95% CI were taken directly from the study. If HR information is not available, we indirectly converted Kaplan–Meier survival curve profiles into x and y coordinates using Engauge Digitizer software to extract time-specific survival rates to convert them into HR and 95% CI [19, 20]. The Cochrane Q test combined with the I2 value were implemented for the evaluation of the heterogeneity between the selected studies. If there was no significant heterogeneity between studies (*P* > 0.1, I2 < 50%), the fixed effects model was used for combinatorial analysis. Otherwise, the random effects model is used [21]. The assessment of publication bias was realized by Begg’s funnel plot along with Egger’s test. R software (version 4.1.2) was implemented for statistical analysis. *P* < 0.05 was defined with statistical significance.

## Results

### Study traits

**Fig. 1:**
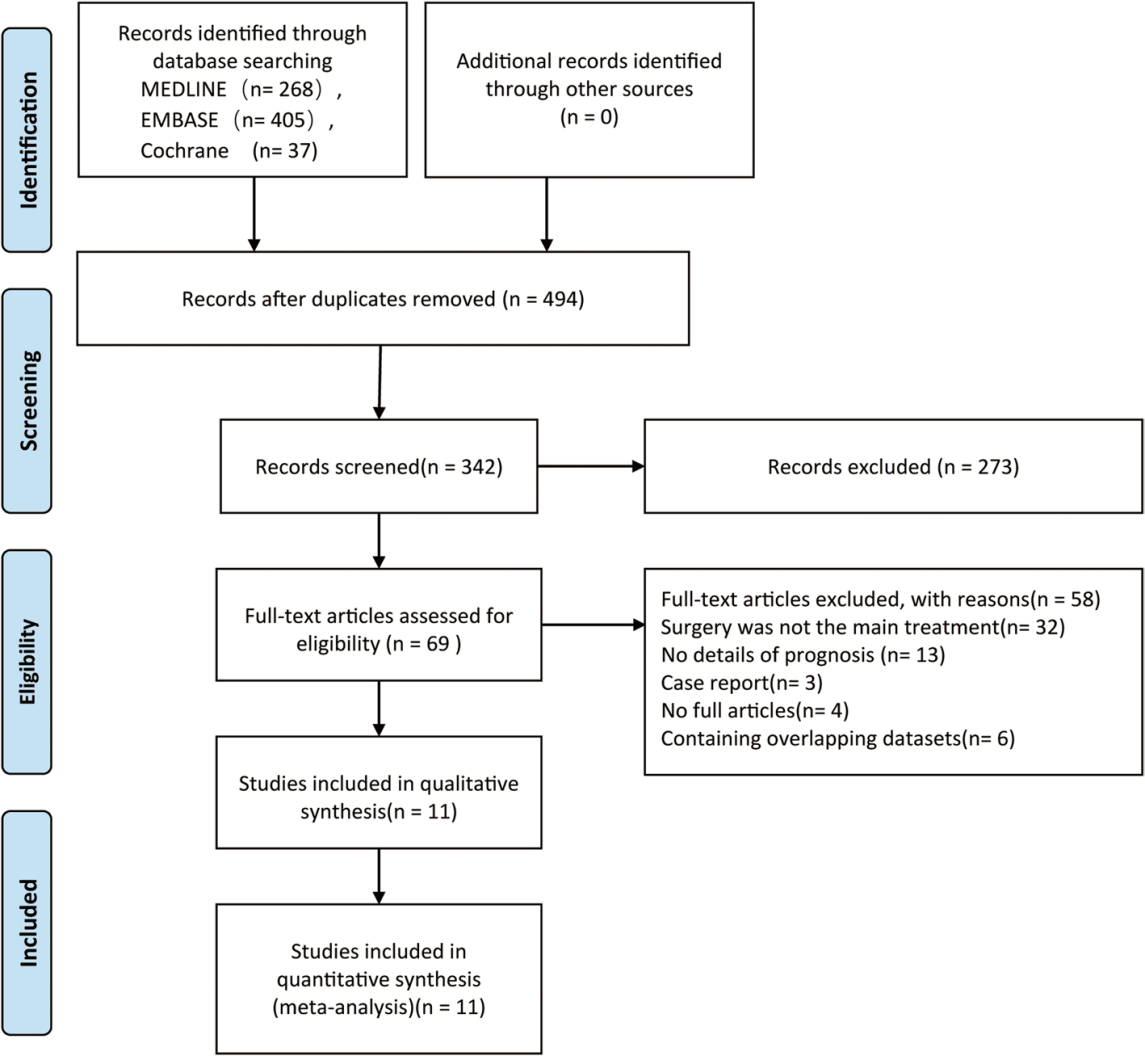
Flow chart of the screening of enrolled studies

Through the search of MEDLINE, EMBASE, and Cochrane databases, 710 potential related studies were retrieved. We have formulated a detailed retrieval flow chart (Fig. 1). Eleven articles, also retrospective studies, published between 2001 and 2021 were qualified in our meta-analysis following inclusion and exclusion criteria, including 1,131 SPLC patients. Tables 2, 3 and 4 summarizes the features of the enrolled publications. Concerning the data of 11 studies [22–32], the 5-year OS rate of the first primary lung cancer was 77% (73–85.2%) and the rate after mSPLC surgery was 51% (37.7–63.4%).

**Table 2:** Clinicopathologic traits of included articles

Study (year)	Period	Region	Cases			Male/Female	Second Intervention Mean Age(years)	Smoker	NOS Scale
			Total	mSPLC	sMPLC				
Hamaji et al.(2013) [31]	2000–2009	USA	161	161	NR	88/73	70	138	7
Yang et al.(2014) [30]	2006–2011	China	143	143	NR	106/37	60	69	7
Zhao et al.(2017) [23]	2001–2014	China	115	115	NR	55/60	60	35	6
Sato et al.(2021) [28]	2005–2017	Japan	61	61	NR	39/22	68.9 ± 1.1	39	7
Doddoli et al.(2001) [27]	1985–1999	France	38	38	NR	35/3	63 ± 8	NR	6
Lee et al.(2009) [32]	1995–2008	USA	58	58	NR	23/35	67	NR	6
Zuin et al.(2013) [24]	1995–2010	Itaty	121	98	23	105/16	68 ± 9.9	NR	5
Aziz et al.(2002) [25]	1986–1999	UK	51	41	10	45/6	64 ± 6.9	51	6
Riquet et al.(2008) [26]	1983–2005	France	234	116	118	194/40	63.7 ± 9.1	NR	7
Battafarano et al.(2004) [22]	1988–2002	USA	69	69	NR	36/33	67.5 ± 8.9	NR	5
Rea et al.(2001) [29]	1971–2002	Italy	80	61	19	72/8	63	NR	6

*NR* Not reported, *NOS* Newcastle–Ottawa scale

**Table 3:** Clinicopathologic traits of included articles (continued)

Study (year)	Extent of resection first operation			Extent of lung resection second operation			Lymph node metastasis		5-year OS (%)	
	Sublobar	Lobectomy	Pneumonectomy	Sublobar	Lobectomy	Completion pneumonectomy	N0	N1 + N2	First tumor	mSPLC
Hamaji et al.(2013) [31]	28	126	7	124	36	1	104	57	87.4	60.8
Yang et al.(2014) [30]	1	140	2	60	50	33	82	61	83.9	54.5
Zhao et al.(2017) [23]	21	94	NR	58	57	NR	87	28	86.5	69.5
Sato et al.(2021) [28]	14	47	NR	39	11	11	41	20	NR	78.7
Doddoli et al.(2001) [27]	NR	36	2	12	10	16	27	11	70	32
Lee et al.(2009) [32]	6	50	2	35	23	NR	56	2	NR	66
Zuin et al.(2013) [24]	10	106	5	60	44	17	107	14	76	42
Aziz et al.(2002) [25]	8	32	1	4	26	11	33	8	NR	44
Riquet et al.(2008) [26]	NR	NR	NR	54	103	77	144	90	NR	31.6
Battafarano et al.(2004) [22]	9	57	3	34	31	4	50	19	60.9	33.4
Rea et al.(2001) [29]	13	65	2	40	32	8	71	9	77	51

*NR* Not reported, *OS* Overall survival

**Table 4:** Fistopathological types of included articles

Study (year)	Histology of first tumor			Histology of second tumor			Histology	
	AC	SCC	other	AC	SCC	other	same	different
Hamaji et al.(2013) [31]	105	37	19	98	41	22	123	38
Yang et al.(2014) [30]	65	56	22	64	54	25	NR	NR
Zhao et al.(2017) [23]	NR	NR	NR	NR	NR	NR	93	22
Sato et al.(2021) [28]	48	11	2	NR	NR	NR	53	8
Doddoli et al.(2001) [27]	14	20	4	14	17	7	23	15
Lee et al.(2009) [32]	47	5	6	48	6	4	42	12
Zuin et al.(2013) [24]	NR	NR	NR	49	38	34	NR	NR
Aziz et al.(2002) [25]	14	32	5	15	24	12	28	23
Riquet et al.(2008) [26]	NR	NR	NR	NR	NR	NR	136	98
Battafarano et al.(2004) [22]	NR	NR	NR	NR	NR	NR	43	26
Rea et al.(2001) [29]	NR	NR	NR	NR	NR	NR	NR	NR

*AC* Adenocarcinoma, *SCC* Squamous cell carcinoma

### Quality estimation and risk of bias

A modified NOS scale was utilized for the quality estimation of the enrolled literature, with the results shown in Table 2. All selected articles had a NOS score of equal to or more than 5 points, suggesting their high quality, which indicated a reduced risk of bias in this study.

### Surgical methods for SPLC

**Fig. 2:**
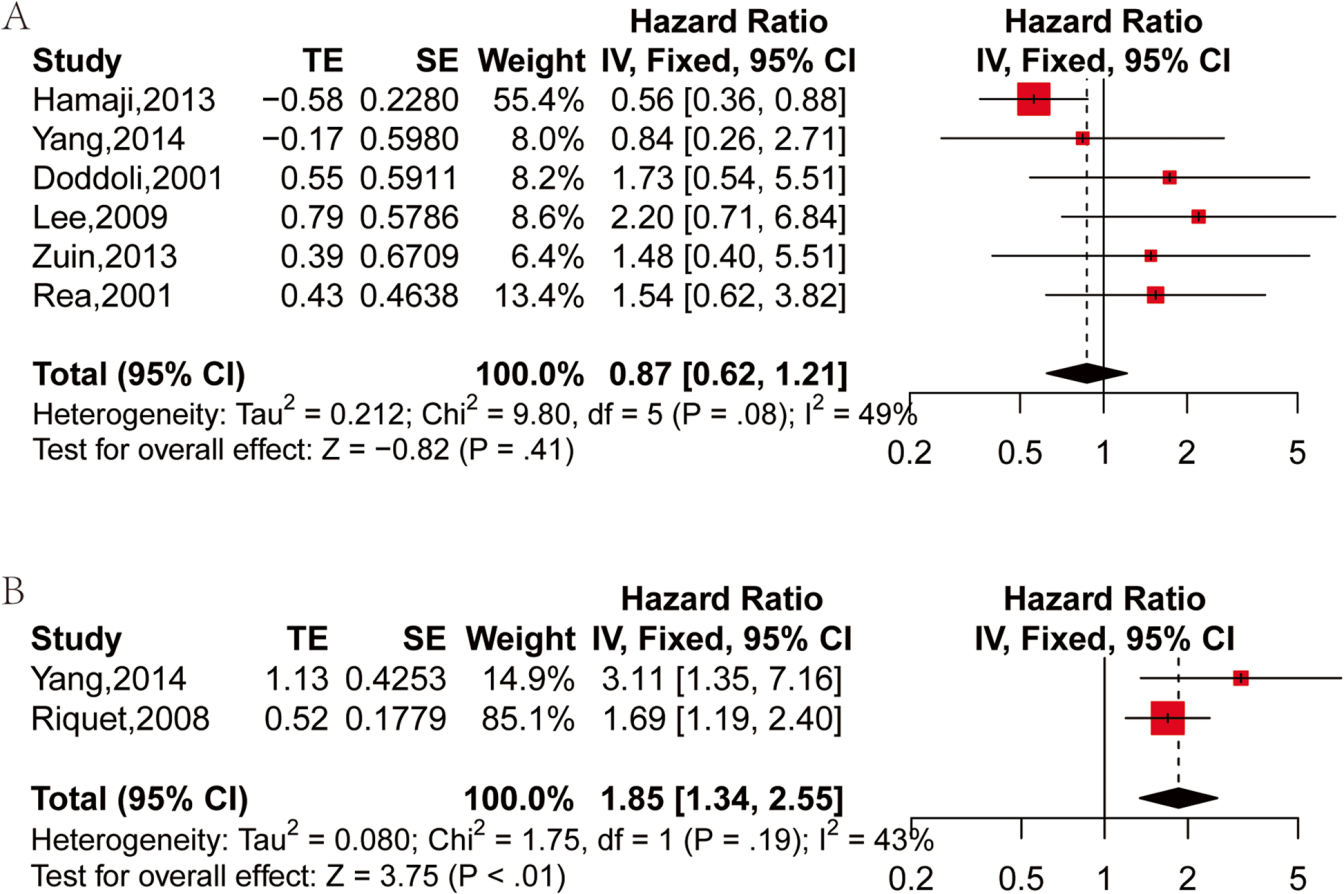
Forest plot of HR of OS for surgery methods: **A** sublobar resection vs lobectomy in SPLC; **B** completion pneumonectomy vs non-completion pneumonectomy in SPLC

The OS of the mSPLC was calculated from the second operation. A total of six studies compared the OS of lobectomy and sublobar resection (segmentectomy or wedge resection) in the treatment of SPLC [24, 27, 29–32], and no significant difference was witnessed in OS after sublobar resection in contrast to lobectomy for SPLC patients (HR: 0.87, 95%CI: 0.62–1.21, *P* = 0.41) by using a fixed effect model (I^2^ = 49%, *P* = 0.08) (Fig. 2A).

Two studies compared the OS of SPLC treated by completion pneumonotomy and non-completion pneumonotomy [26, 30], and patients who received completion pneumonectomy had worse OS in comparison to those who received non-completion pneumonectomy (HR: 1.85, 95%CI: 1.34–2.55, *P* < 0.01) by using the fixed effects model (I^2^ = 43%, *P* = 0.19) (Fig. 2B).

### sSPLC vs mSPLC

The part aimed to investigate OS starting from the first and second tumor operations for mSPLC and compare the prognosis between sSPLC and mSPLC.

**Fig. 3:**
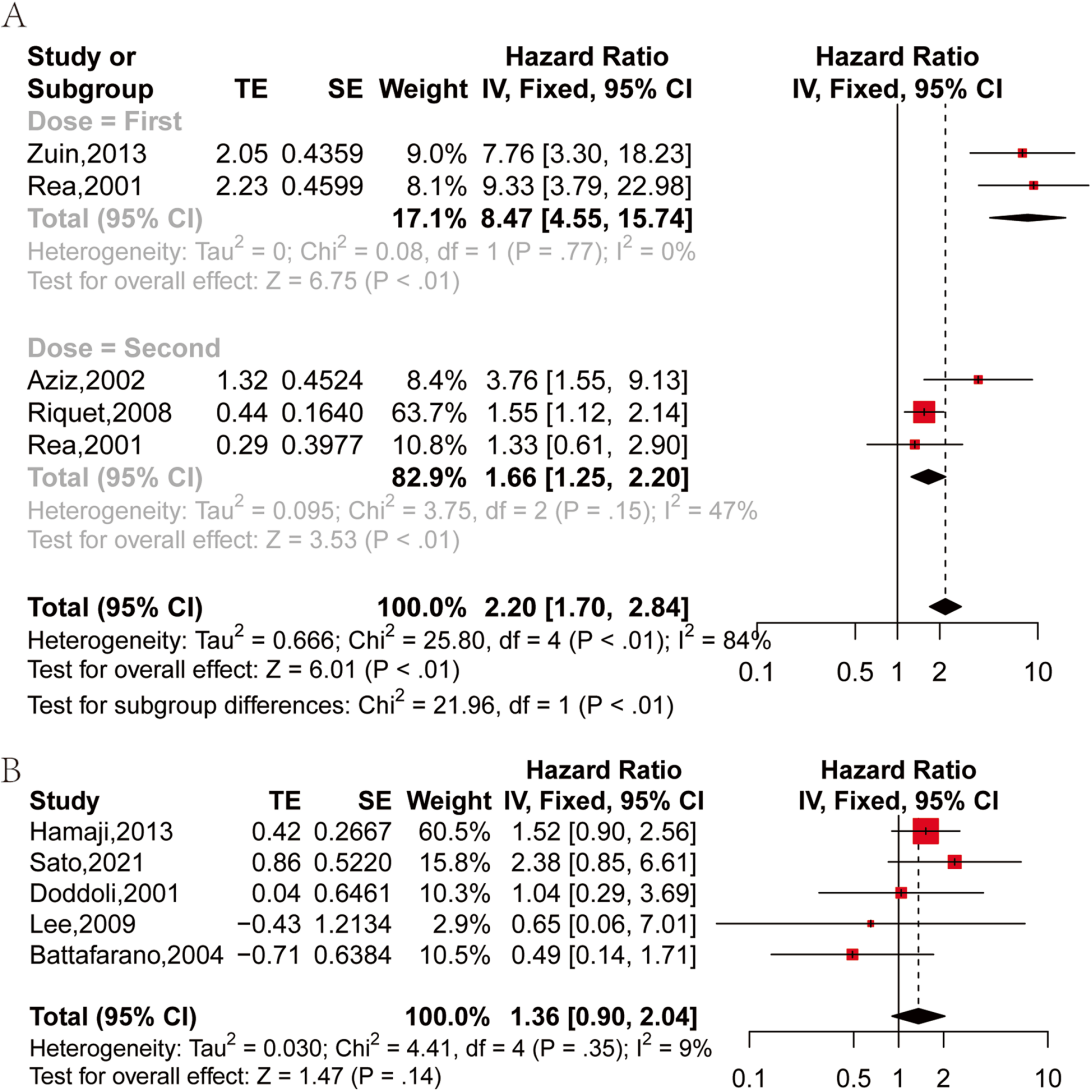
Forest plot of HR of OS: **A** sSPLC vs mSPLC. **B** DFI ≥ 2 years vs DFI < 2 years

The OS of the mSPLC was calculated from the first primary tumor operation. There were two studies comparing the OS of sSPLC and mSPLC [24, 29]. There was a ldecreased OS in patients with sSPLC in comparison to those with mSPLC (HR: 8.47, 95% CI: 4.55–15.74, *P* < 0.01) by using the fixed effect model (I^2^ = 0%, *P* = 0.77) (Fig. 3A: First).

The OS of mSPLC was calculated from the second primary tumor surgery. There were three studies comparing the OS of sSPLC and mSPLC [25, 26, 29]. A lower OS was also observed in sSPLC patients in contrast to the mSPLC patients (HR: 1.66, 95% CI: 1.25–2.20, *P* < 0.01) using the fixed effect model (I^2^ = 47%, *P* = 0.15) (Fig. 3A: Second).

### The OS of MSPLC based on the disease-free interval(DFI)

There were 5 studies that compare OS difference between DFI less than or greater than 2 years [22, 27, 28, 31, 32]. HR and 95% CI of SPLC patients with DFI ≥ 2 years and DFI < 2 years were extracted or calculated from each study. Using the fixed effect model (I^2^ = 9%, *P* = 0.35), no marked difference was witnessed in OS between patients with DFI ≥ 2 years and those with DFI < 2 years (HR: 1.36, 95% CI: 0.90–2.04, *P* = 0.14) (Fig. 3B).

### Similarity and difference in histology in primary and second primary tumors

**Fig. 4:**
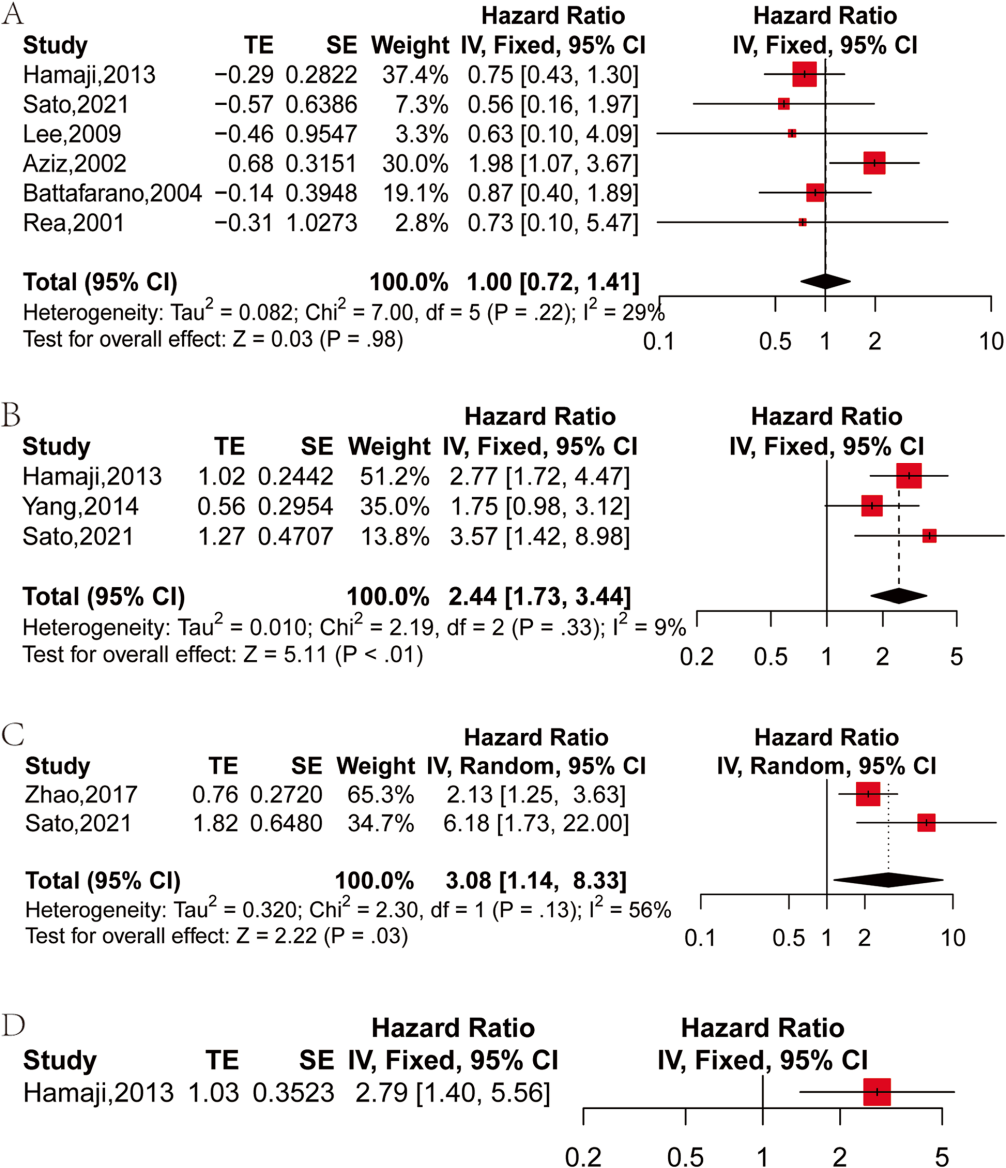
Forest plot of HR of OS for prognostic factors: **A** similarity and difference in histology between primary and second primary tumors; **B** tumor size of SPLC > 2 cm vs ≤ 2 cm; **C** solid-p vs GGO-p in CT morphology; **D** node positive vs node negative

Six studies have compared histological similarities and differences in OS between primary and secondary primary tumors [22, 25, 28, 29, 31, 32]. From each study, HR and 95% CI of OS were extracted or calculated in patients with primary lung cancer and SPLC with the same or different histologies. The histological similarities and differences exhibited no difference in OS between primary lung cancer and SPLC (HR: 1.00, 95% CI: 0.72–1.41, *P* = 0.98) by using the fixed effect model (I^2^ = 29%, *P* = 0.22) (Fig. 4A).

### Tumor size

Three studies have compared the influence of SPLC tumor size on OS [28, 30, 31]. From each study, HR and 95% CI of OS for SPLC patients with tumor size > 2 cm and ≤ 2 cm were extracted or calculated. There was worse OS of SPLC patients with tumor size of > 2 cm in contrasct to those with tumor size ≤ 2 cm (HR: 2.44, 95%CI: 1.73–3.44, *P* < 0.01) by using the fixed-effect model (I^2^ = 9%, *P* = 0.33) (Fig. 4B).

### CT morphology

Following the CT morphology and consolidation/tumor ratio (CTR), SPLCs were subsequently classified into solid predominant (Solid-p; containing tumors with CTR > 50% and pure solid) and ground glass opacity predominant (GGO-p; containing pure GGO and CTR ≤ 50% GGO). Two studies have compared the difference between Solid-p and GGO-p in the CT morphology of SPLC patients [23, 28]. Using the random effect model (I^2^ = 56%, *P* = 0.13), different CT appearances of SPLC disclosed that there was a worse OS of Solid-p patients versus that of GGO-p patients (HR: 3.08, 95% CI: 1.14–8.33, *P* = 0.03) (Fig. 4C).

### LNM status

The HR and 95% CI of OS in LNM-positive and negative patients were only extracted or calculated from one study [31]. Using the fixed-effect model, LNM-positive patients harbored a worse OS versus LNM-negative patients (HR: 2.79, 95%CI: 1.40–5.56) (Fig. 4D).

### Smoking status

**Fig. 5:**
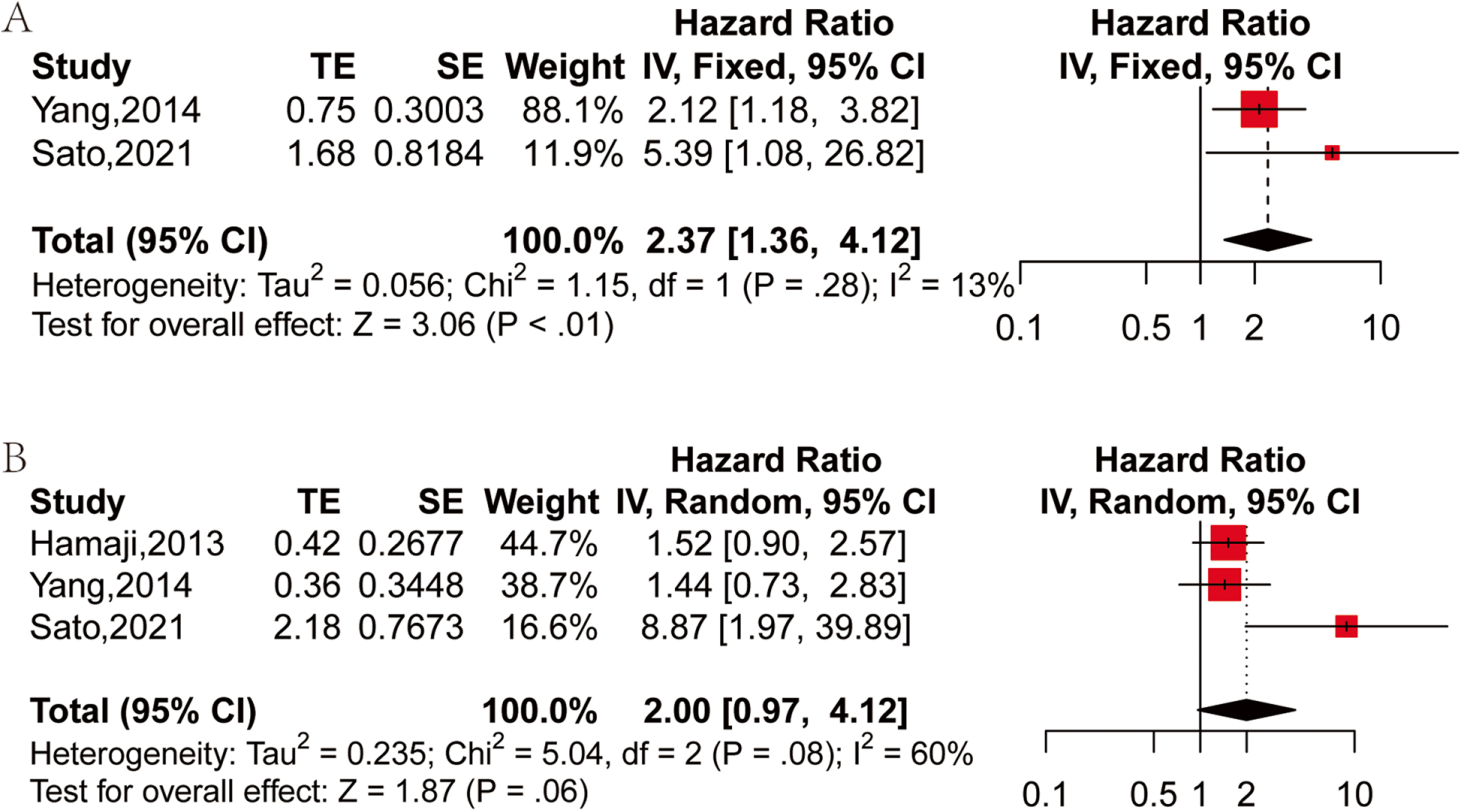
Forest plot of HR of OS for prognostic factors: **A** smokers vs non-smokers; **B** male vs female

Two studies have compared the OS of smokers and non-smokers in SPLC patients [28, 30]. From each study, HR and 95% CI of the OS of the smokers and non-smokers were extracted or calculated. Using the fixed effect model (I^2^ = 13%, *P* = 0.13), there exhibited a lower OS of the smokers in SPLC patients in contrast to that of the non-smokers (HR: 2.37, 95% CI: 1.08–26.82, *P* < 0.01) (Fig. 5A).

### Gender

Three studies compared the effects of male and female SPLC patients on OS [28, 30, 31]. The extraction or calculation of HR and 95% CI for OS of male and female patients was conducted in each study. No difference was witnessed in OS between the genders of SPLC patients (HR: 2.00, 95%CI: 0.97–4.12, *P* = 0.06) with a random-effects model (I^2^ = 60%, *P* = 0.08) (Fig. 5B).

### Publication bias

Since there were fewer than 10 studies for each prognostic factor in this meta-analysis, funnel plots could not be implemented for the estimation of publication bias. The publication bias was assessed by the Egger test for each prognostic factor, and no marked publication bias was witnessed in the evaluation of SPLC type, DFI, pathological type, tumor size, gender, etc. (all *P* > 0.05). Publication bias was found in SPLC surgery (*P* < 0.05).

## Discussion

SPLC, as a distinctive type of lung cancer, is independent of primary lung cancer. Multiple publications have disclosed that SPLC patients harbor a better prognostic outcomes than those with local recurrence or metastasis [33–35]. The eighth edition lung cancer staging system does not distinguish between SPLC and IM, resulting in the failure to truly evaluate the patient's condition and a delay in treatment. Currently, there are no standards for the diagnosis and therapy of SPLC, and the strategies remain controversial for these aspects [13]. Surgery remains the first-line treatment for SPLC. This systematic review and meta-analysis first evaluated the prognostic factors after SPLC surgery, involving 11 studies (including 1,131 patients).

As mentioned before, surgery is the preferred treatment for SPLC. Nevertheless, a unified surgical strategy for SPLC has not been established due to the lack of evidence-based medical evidence such as related clinical trials. At present, the extent of surgical resection of SPLC mainly depends on the status and tumor characteristics of patients [24, 36]. Sublobar resection includes segmentectomy and wedge resection. Chen et al. [37] supported that the OS of SPLC patients received sublobar resection was comparable to those received lobectomy. However, this study included therapeutic method of primary lung cancer, and not all patients received surgical treatment for SPLC. In this research, the patients with the first primary lung cancer were all received surgical resection.

In SPLC treatment, the findings of this study unveiled that sublobar resection can replace lobectomy without affecting OS. The same conclusion was obtained in patients with mSPLC. Therefore, sublobar resection provides a better choice for patients whose cardiopulmonary function cannot tolerate the second operation. Meanwhile, this study also found that completion pneumonectomy is a poor prognostic factor due to the high risk of surgery and the high incidence of postoperative complications, which should be avoided by SPLC patients. Therefore, for the selection of surgical methods for SPLC patients, more lung functions should be retained as far as possible according to the actual situation of patients, and more accurate surgical evaluation should be performed with the help of experienced multidisciplinary teams [38, 39].

SPLCs were classified into sSPLCs and mSPLCs based on the time of lesion occurrence. The starting point of the study on the survival rate of mSPLC is controversial due to the existence of the DFI of mSPLC. Rosengart et al. [40] believed that the alleviation of OS may be related to the increased time interval between primary lung cancer and mSPLC. Through this meta-analysis, we observed that the OS in mSPLC patients outperformed that of sSPLC patients regardless of OS counted from the operation of primary lung cancer or SPLC. This is not consistent with the findings obtained from the systematic review and meta-analysis by Jiang et al. [41]. This research concluded that no difference was witnessed in OS between mSPLC and sSPLC patients when OS was calculated from the start of SPLC surgery. Therefore, more research is warranted for further verification.

Currently, the generally accepted diagnostic standards for SPLC was put forward by Martini and Melamed [6]. The recommended diagnostic criteria are at least 2 years of SPLC free interval between cancers. In 2013, the American College of Chest Physicians (ACCP) updated the diagnostic standards to change the diagnostic interval to 4 years between mSPLC and primary lung cancer [13]. Unfortunately, this standard has not been widely accepted, and most current studies still recommend 2 years as the standard for distinguishing between sSPLC and mSPLC. This meta-analysis revealed no marked difference in OS between patients with DFI ≥ 2 years and those with less than 2 years. This conclusion indicates that with the deepening understanding of SPLC, it is worth further discussion on whether to continue to use DFI ≥ 2 years as the time to distinguish mSPLCs and sSPLCs.

Although TNM staging of lung cancer is vital to the prognosis of patients with lung cancer, the SPLC included in this article is evaluated by histology, tumor size, LNM status, as well as CT morphology of SPLC because it cannot be evaluated according to the unified TNM staging. This meta-analysis disclosed that the same or different histological subtypes between primary and second primary tumors had no effect on the prognosis. However, tumor diameter > 2 cm, solid predominant in CT morphology, and LNM were considered to be poor prognostic factors for SPLC. On account of the diagnostic standards of Martini and Melamed [6], SPLC can present the same or diverse histology as the primary tumor. When the histology is the same or similar, the following aspects shall be met to distinguish primary cancer from IM: SPLC development of the novel lesion from an in situ carcinoma; Neither had a common lymphangiocarcinoma; no extra-pulmonary metastasis during diagnosis. Many studies have found that molecular analysis methods (including TP53 mutation analysis, DNA microsatellite analysis, genomic breakpoint analysis, and especially NGS) are used to identify SPLC and IM [42–45]. After the exclusion of IM, the primary lung cancer and SPLC had relatively independent biological characteristics, and thus different/identical histological types or subtypes had no significant effect on prognosis. Similarly, the impacts of tumor size, CT morphology, and LNM status on the prognosis of SPLC should be similar to those of primary lung cancer. The results of this work also confirm this view. In related studies of primary lung cancer, it has been found that solid predominant (solid-p) has more malignant potential (eg. vascular infiltration or LNM) than ground glass opacity predominant (GGO-p), even if the tumor size is ≤ 2 cm [46]. In other words, CT morphologic GGO-p has a greater survival advantage than solid-p. This is the same conclusion as found in the meta-analysis of CT morphology in SPLC in this study. Recent studies (JCOG0802/WJOG4607L) [47] have shown that patients with early peripheral NSCLC (tumor diameter ≤ 2 cm, CTR > 50%) harbor a high 5-year OS rate of segmentectomy versus that of lobotomy, but the local recurrence rate of segmentectomy is significantly higher than lobotomy. However, there is no similar study on SPLC. Meanwhile, the need for postoperative adjuvant therapy in SPLC patients with large tumors and LNMs also deserves further discussion.

This meta-analysis also demonstrated that gender differences did not affect the survival rate of SPLC patients. Smoking is an adverse factor for influencing the prognosis of SPLC patients. Aredo et al. [48] found that smoking is regarded as a risk factor for SPLC among survivors of primary lung cancer, which is the same as our conclusion. Besides, this study also unveiled that the risk of SPLC in patients smoking after early primary lung cancer treatment has an elevated risk of SPLC compared to advanced primary lung cancer. Therefore, it is an effective prevention strategy for SPLC to actively quit smoking for primary lung cancer patients, and to conduct SPLC monitoring for high-risk patients (eg. active smokers and early primary lung cancer) during diagnosis [4].

From the above studies, it can be inferred that SPLC has relatively independent biological characteristics, which is not associated with the first primary lung cancer. Therefore, SPLC has similar prognostic characteristics with single primary lung cancer, providing a basis for assessing the T, N, M staging separately for each lesion of multiple primary lung cancer and guiding SPLC treatment.

Limitations are also found in our meta-analysis. First of all, all the selected studies were retrospective studies, and there may be selection bias. The propensity score matching was not implemented to eliminate the impacts of other factors on the observation findings. Secondly, this meta-analysis only includes articles published in English, which inevitably increases publication bias. Third, there is publication bias in the surgical method due to the long time span with the inclusion of the study. The surgical effect is affected by the technology and the surgeon level. Additionally, according to this meta-analysis, the OS after sSPLC is superior to mSPLC, which may lead to publication bias. Fourth, prognostic factors are included in the study with a small sample size so conclusions should refer carefully.

## Conclusion

In summary, this meta-analysis highlights that sublobar resection should be given priority for SPLC patients, especially in those with oor cardiopulmonary function because of the similar prognosis between sublobar resection and lobectomy. SPLC patients should try to avoid completion pneumonectomy. Patients with sSPLC, tumor diameter > 2 cm, solid predominant CT morphology, smoking, and LNM had a poor prognosis. This research provides a basis for surgical treatment of SPLC. Besides, SPLC has similar prognostic characteristics with single primary lung cancer, which offers a basis for evaluating the T, N, M staging separately for each lesion of multiple primary lung cancer. However, the study has some limitations and more research is warranted to verify the conclusion.

## Data Availability

The datasets generated and analyzed during the current study are available from the corresponding author on reasonable request.
